# Current advances in the epidemiology, risk factors, prevention, management, and long-term outcomes of herpes zoster and post-herpetic neuralgia

**DOI:** 10.3389/fmicb.2026.1903334

**Published:** 2026-08-19

**Authors:** Anzah Imtiaz Waggan, Kinzah Imtiaz, Oluwatoyin Ayo-Farai, Harry Okwilagwe, Iyoha Ehi-Iyoha, Riaz Akhtar, Malik Olatunde Oduoye, Mohammed Quader Naseer

**Affiliations:** 1 Department of Medicine, Jinnah Sindh Medical University, Karachi, Pakistan; 2 Department of Medicine, Dow University Hospital, Karachi, Pakistan; 3 College of Public Health, Georgia Southern University, Statesboro, GA, United States; 4 College of Medicine, Ambrose Alli University, Ekpoma, Edo, Nigeria; 5 Department of Medicine, Bacha Khan Medical College, Mardan, Pakistan; 6 Department of Research, The Medical Research Circle (MedReC), Goma, Democratic Republic of Congo; 7 Department of Medicine, Ayaan Institute of Medical Sciences, Hyderabad, India

**Keywords:** herpes zoster, management, neuropathic pain, outcomes, pain control, post-herpetic neuralgia, risk factors, shingles

## Abstract

**Background:**

Herpes zoster (HZ) results from reactivation of latent varicella-zoster virus and is a major cause of morbidity, particularly among older adults and immunocompromised individuals. Post-herpetic neuralgia (PHN), the most common chronic complication of HZ, is characterized by persistent neuropathic pain that can substantially impair quality of life. Despite advances in antiviral therapy and vaccination, PHN remains a significant clinical challenge. This review presents current evidence on the epidemiology, risk factors, pathophysiology, clinical manifestations, prevention, and management of HZ and PHN, with emphasis on factors associated with PHN development and contemporary therapeutic approaches.

**Methods:**

A narrative review of the literature was conducted using publications indexed in PubMed, Scopus, Web of Science, and Google Scholar from January 2010 to June 2026. Peer-reviewed studies, systematic reviews, meta-analyses, randomized controlled trials, cohort studies, and clinical guidelines addressing HZ, PHN, and related management strategies were included. Findings were synthesized narratively.

**Results:**

Advanced age, severe acute zoster pain, extensive rash involvement, prodromal pain, and immunocompromised status were consistently identified as major predictors of PHN. Early initiation of antiviral therapy within 72 h of rash onset was associated with reduced acute disease severity and may decrease the risk of prolonged pain. Evidence supports the use of gabapentinoids, tricyclic antidepressants, topical lidocaine, and capsaicin for PHN management, while multidisciplinary approaches incorporating non-pharmacologic interventions may further improve outcomes. Recombinant zoster vaccination demonstrates high effectiveness in preventing HZ and PHN in older adults.

**Conclusion:**

PHN remains a substantial source of long-term morbidity following HZ, particularly in aging populations. Early recognition of high-risk patients, prompt antiviral treatment, evidence-based pain management, and wider uptake of recombinant zoster vaccination are key strategies for reducing disease burden and improving patient outcomes.

## Highlights

- This review synthesizes recent evidence on the epidemiology, risk factors, management, and long-term outcomes of herpes zoster and post-herpetic neuralgia.
- Advanced age, severe acute zoster pain, extensive rash, and immunocompromised status are consistently identified as the strongest predictors of post-herpetic neuralgia.
- Early initiation of antiviral therapy and timely multimodal pain management may reduce disease severity and improve long-term patient outcomes, although they do not completely prevent post-herpetic neuralgia.
- Recombinant zoster vaccination remains the most effective strategy for preventing herpes zoster and its long-term complications, yet vaccine uptake remains suboptimal, particularly in low- and middle-income countries.
- Integrating evidence-based pharmacological, non-pharmacological, and preventive strategies can improve quality of life and reduce the long-term clinical and economic burden of post-herpetic neuralgia.

## 1 Introduction

Herpes zoster (HZ), commonly known as shingles, is a neurocutaneous disease caused by the reactivation of latent varicella-zoster virus (VZV), a double-stranded DNA virus belonging to the *Herpesviridae* family (Fan et al., 2023). Following primary infection, which usually presents as varicella (chickenpox), VZV establishes lifelong latency within the dorsal root and cranial nerve ganglia (Gilden et al., 2011). Reactivation occurs when VZV-specific cell-mediated immunity declines, most commonly due to aging, immunosuppression, malignancy, HIV infection, organ transplantation, or the use of immunosuppressive medications (Son et al., 2025).

HZ remains an important global public health problem because of its increasing incidence in aging populations and individuals with impaired immune function (Curran et al., 2023). Approximately one in three people will develop HZ during their lifetime (Wong and Levin, 2019). Although HZ affects individuals worldwide, disease burden is disproportionately higher among older adults owing to immunosenescence and among immunocompromised individuals because of their increased susceptibility to viral reactivation and disease complications (John and Canaday, 2017; McKay et al., 2020; Wu et al., 2025).

The characteristic clinical presentation of herpes zoster is a unilateral, painful vesicular eruption confined to one or more dermatomes (Patil et al., 2022; Lim et al., 2024). The rash is frequently preceded by prodromal symptoms, including localized pain, burning sensation, itching, paraesthesia, or hyperesthesia occurring several days before the appearance of skin lesions (CDC, 2024). Although the cutaneous manifestations usually resolve within 2 to 4 weeks, persistent neuropathic pain may continue long after the rash has healed, resulting in post-herpetic neuralgia (PHN), the most common chronic complication of herpes zoster (Johnson et al., 2010).

PHN is generally defined as pain persisting for at least 90 days after rash onset, although different definitions have been adopted in clinical studies (Tsubaki et al., 2024). PHN is characterized by persistent neuropathic pain, including burning pain, stabbing sensations, electric shock-like pain, mechanical allodynia, hyperalgesia, and spontaneous pain (Liu Q. et al., 2024). The condition is associated with substantial impairment in physical functioning, sleep quality, emotional wellbeing, social participation, and overall health-related quality of life (QoL) (Liu Y. et al., 2024). Furthermore, PHN contributes significantly to healthcare utilization, prolonged treatment, and economic burden, particularly among older adults (Serpell et al., 2014; Ogunyemi et al., 2025).

Several clinical and demographic factors have consistently been identified as predictors of PHN (Forbes et al., 2016a,b). Advanced age remains the strongest risk factor because of progressive decline in VZV-specific cell-mediated immunity (Forbes et al., 2016a,b). Other recognized predictors include severe acute zoster pain, extensive rash, ophthalmic involvement, prodromal pain, delayed initiation of antiviral therapy, and immunocompromised status (Litt et al., 2024). Early identification of these high-risk individuals enables timely intervention and may reduce disease-related complications (CDC, 2024).

Prompt antiviral therapy remains the cornerstone of acute herpes zoster management. Antiviral agents, including acyclovir, valacyclovir, and famciclovir, are most effective when initiated within 72 h of rash onset (Chen et al., 2014). Early treatment suppresses viral replication, accelerates healing of skin lesions, reduces the severity and duration of acute pain, and may decrease the risk of prolonged neuropathic pain (Bruxelle and Pinchinat, 2012). However, antiviral therapy alone does not completely prevent the development of PHN, highlighting the need for comprehensive pain management strategies (Rabaud et al., 2013).

The management of PHN remains challenging because of its chronic neuropathic nature and the variable response to treatment among affected individuals (Tontodonati et al., 2012). Current evidence supports a multimodal approach involving gabapentinoids, tricyclic antidepressants, topical lidocaine patches, high-concentration capsaicin patches, and selected interventional pain management techniques for refractory cases (Gudin et al., 2019). Treatment should be individualized according to patient characteristics, pain severity, comorbidities, and medication tolerability, with the primary objectives of reducing pain intensity, improving functional status, and enhancing QoL (Liu Q. et al., 2024).

Vaccination represents the most effective preventive strategy against herpes zoster and its complications (Chiavarini et al., 2025). The recombinant zoster vaccine has demonstrated high efficacy in preventing both herpes zoster and PHN among immunocompetent older adults and selected immunocompromised populations (Chiavarini et al., 2025; Bibera et al., 2024). Consequently, many national immunization programmes now recommend recombinant zoster vaccination for adults aged 50 years and older and for high-risk individuals (Chiavarini et al., 2025). Nevertheless, disparities in vaccine availability and uptake continue to limit the full public health benefits of vaccination, particularly in low- and middle-income countries (LMICs) (Bibera et al., 2024; Varghese and Mishra, 2026; Bagattini et al., 2025).

Despite considerable advances in understanding the biology, prevention, and treatment of HZ, PHN continues to represent a significant cause of chronic pain and disability worldwide (Liang et al., 2025). Population aging, increasing prevalence of immunocompromising conditions, and gaps in vaccine implementation underscore the need for continuous evaluation of emerging evidence. A comprehensive synthesis of current knowledge is therefore essential to support evidence-based clinical practice and improve patient outcomes.

Accordingly, this study summarizes current evidence on the epidemiology, pathophysiology, clinical manifestations, predictors of post-herpetic neuralgia, preventive strategies, and contemporary management of herpes zoster, with particular emphasis on recent advances that may enhance early diagnosis, optimize treatment, improve quality of life (QoL), and reduce the long-term burden of disease.

## 2 Methodology

### 2.1 Study design

This study was conducted as a narrative literature review to summarize and critically synthesize current evidence on the epidemiology, pathophysiology, risk factors, clinical manifestations, prevention, and management of herpes zoster (HZ) and post-herpetic neuralgia (PHN). A narrative review design was considered appropriate because the objective was to provide a comprehensive overview of contemporary evidence and emerging developments rather than to quantitatively pool study findings.

### 2.2 Literature search strategy

A comprehensive literature search was undertaken using the electronic databases PubMed/MEDLINE, Scopus, Web of Science, Google Scholar, and the Cochrane Library. The search included articles published from January 2010 to June 2026 to capture recent advances in the epidemiology, prevention, diagnosis, and management of HZ and PHN.

The search strategy combined Medical Subject Headings (MeSH) and free-text keywords using Boolean operators (“AND” and “OR”). The principal search terms included: “Herpes zoster”, “Shingles”, “Varicella-zoster virus”, “Post-herpetic neuralgia”, “Neuropathic pain”, “Risk factors”, “Epidemiology”, “Antiviral therapy”, “Vaccination”, “Management”. Reference lists of relevant review articles, clinical guidelines, and original research publications were also manually screened to identify additional eligible studies that were not retrieved during the electronic database search.

### 2.3 Eligibility criteria

Peer-reviewed publications addressing one or more aspects of herpes zoster or post-herpetic neuralgia were considered eligible for inclusion. These included randomized controlled trials, cohort studies, case-control studies, cross-sectional studies, systematic reviews, meta-analyses, narrative reviews, evidence-based clinical guidelines, and consensus statements published in English.

Studies were included if they reported information on the epidemiology, pathophysiology, clinical presentation, risk factors, prevention, diagnosis, treatment, vaccination, or long-term outcomes of HZ or PHN. Studies were excluded if they were conference abstracts without full-text publications, editorials lacking primary scientific content, letters to the editor, duplicate publications, animal studies without direct clinical relevance, or articles published in languages other than English.

### 2.4 Study selection

Titles and abstracts retrieved from the literature search were initially reviewed for relevance to the objectives of this review. Full-text articles considered potentially eligible were subsequently examined to determine their suitability for inclusion. Relevant information was extracted from each study, including study characteristics, population, methodology, principal findings, and conclusions relating to herpes zoster and post-herpetic neuralgia.

Priority was given to high-quality evidence, including systematic reviews, meta-analyses, randomized controlled trials, large observational studies, and recommendations from internationally recognized professional organizations.

### 2.5 Methodological note

Although this review is presented as a narrative synthesis, the methodology incorporates several elements commonly associated with systematic reviews, including structured database searches, defined eligibility criteria, and explicit study selection procedures. This hybrid approach was intentionally adopted to enhance rigor and transparency while maintaining the flexibility of a narrative review. By combining systematic search features with narrative synthesis, the review provides a comprehensive yet interpretive overview of current evidence on herpes zoster and post-herpetic neuralgia, rather than a quantitative meta-analysis. This distinction ensures clarity for readers and appropriately situates the work within the spectrum of review methodologies.

## 3 Result

### 3.1 Etiopathogenesis of HZ

#### Primary varicella zoster infection

**Figure 1:**
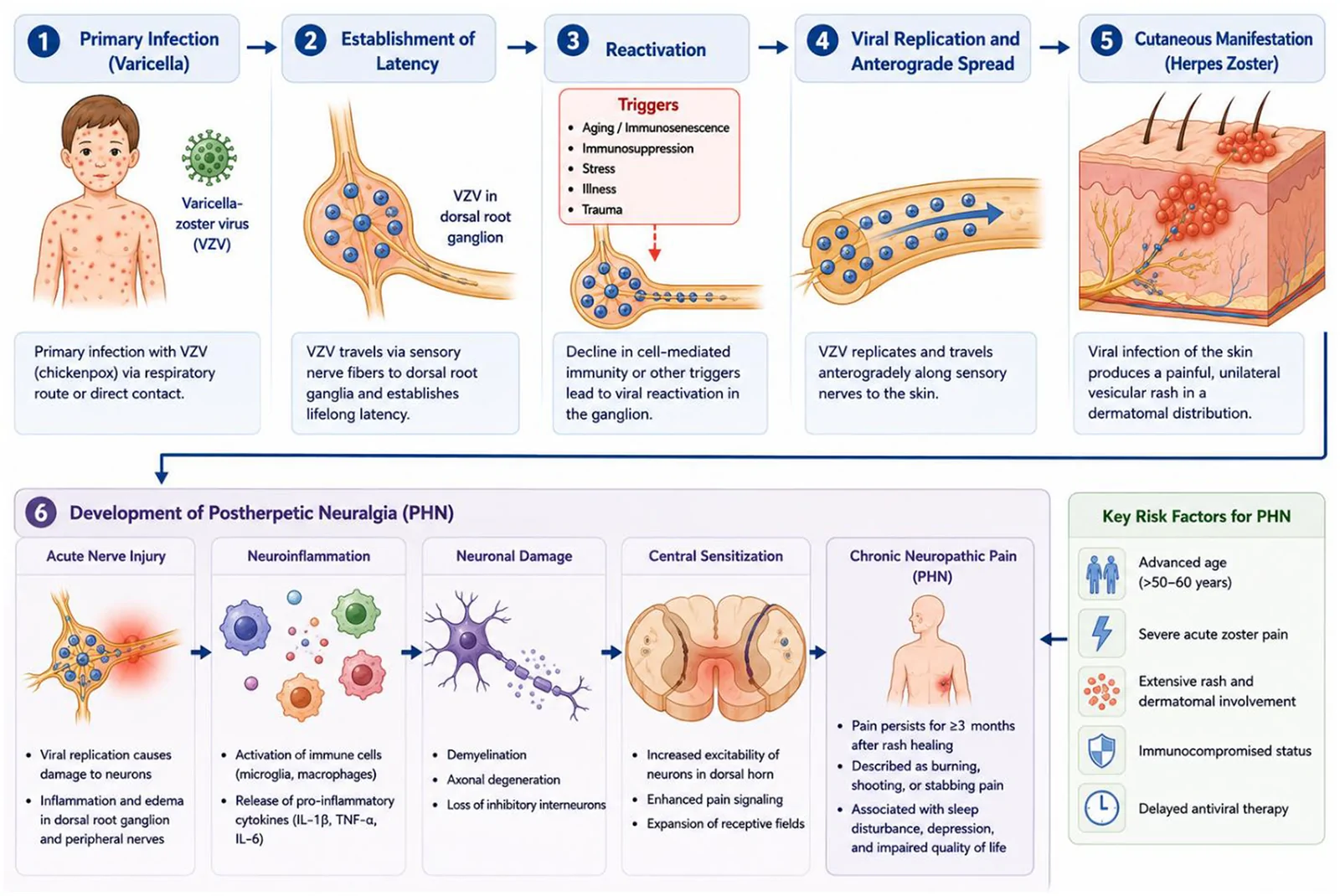
Pathogenesis of herpes zoster and development of PHN.

The transmission of Varicella Zoster virus (Figure 1) is through respiratory droplets or direct contact with vesicular fluids (Patil et al., 2022). It initially affects the epithelial cells of the respiratory tract, from where it gains access to the immune cells in the tonsils and lymphoid tissue, leading to viremia and dissemination to the skin, resulting in the characteristic vesicular rash of chickenpox (Gerada et al., 2020). The virus then enters the latency phase by migrating in a retrograde manner to the neurons of the sensory ganglia. Without active replication, the viral genome remains within the nuclei of neurons (Verzosa et al., 2021).

#### 3.1.2 Latency in sensory ganglia

The viral immediate-early promoter known as ORF63, which remains expressed in the neurons, helps the virus to remain dormant in the sensory ganglia. This phase, which is sustained by the cell-mediated immune system, especially T-cells, can last for decades without causing active disease in the host (Verzosa et al., 2021).

#### 3.1.3 Reactivation of the latent VZV

Reactivation is triggered by aging (as immunity wanes with age) and immunosuppressive conditions (e.g., HIV, cancers, organ transplants, usage of steroids) (Kennedy and Mogensen, 2020). Due to the reduced surveillance by these T-cells, the virus can replicate, causing inflammation in the affected nerve. It then spreads anterogradely through the sensory nerves from the ganglia to the skin (Sutter et al., 2025).

#### 3.1.4 Inflammatory response and nerve damage

Upon reactivation, VZV replicates in the sensory ganglion (Sorel and Messaoudi, 2018). To limit its replication, CD4+ and CD8+ T-cells infiltrate the sensory ganglion, inflicting inflammation and nerve damage. This nerve injury is manifested as excruciating pain, typically of Herpes Zoster, and persists as Post-herpetic neuralgia after the HZ-associated rash resolves (Sutherland et al., 2019).

#### 3.1.5 Clinical manifestations and outcomes

Herpes Zoster's characteristic rash is manifested as a vesicular eruption in a dermatomal pattern, which is usually unilateral, followed by neuropathic pain (Lim et al., 2024). While the infection often resolves on its own in immunocompetent individuals, complications such as disseminated infection, herpes zoster ophthalmicus, or bacterial superinfection of lesions can occur in immunosuppressed people (Litt et al., 2024).

Individuals with active herpes zoster may transmit VZV through direct contact with vesicular fluid to susceptible individuals who lack immunity, resulting in primary varicella (chickenpox) rather than HZ. Airborne transmission is uncommon but may occur in cases of disseminated herpes zoster (Gnann, 2022).

### 3.2 Epidemiology of HZ

HZ is a common viral disease that occurs worldwide and represents a substantial cause of morbidity, particularly among older adults and individuals with impaired cell-mediated immunity. Because VZV establishes lifelong latency following primary varicella infection, virtually all individuals with prior exposure to VZV remain at risk of developing HZ later in life. Lifetime risk is estimated at approximately 30%, rising to nearly 50% among individuals who survive to age 85 (John and Canaday, 2017; Curran et al., 2019).

#### 3.2.1 Demographic trends

The annual incidence of HZ in the general population ranges from 3 to 5 cases per 1,000 person-years, rising to 8–12 cases per 1,000 person-years among adults aged 60 years and older (Kawai et al., 2014). Incidence increases progressively with age due to immunosenescence, which leads to a decline in VZV-specific cell-mediated immunity. Consequently, adults aged 50 years and above account for the majority of HZ cases and experience higher rates of hospitalization, complications, and mortality than younger individuals (Gudin et al., 2019; Marcum et al., 2024; Adachi-Katayama et al., 2026).

**Figure 2:**
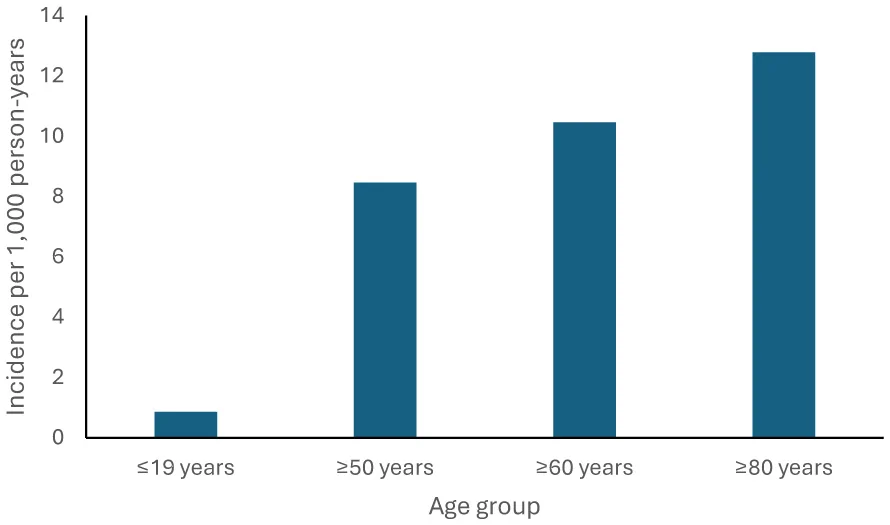
Age-specific incidence of HZ among immunocompetent individuals.

A population-based study of immunocompetent adults in the United States reported an overall HZ incidence of 4.47 per 1,000 person-years, increasing from 0.86 per 1,000 person-years among individuals aged ≤ 19 years to 12.78 per 1,000 person-years in those aged ≥80 years (Johnson et al., 2015). The incidence was 8.46 per 1,000 person-years among adults aged ≥50 years and 10.46 per 1,000 person-years among those aged ≥60 years (Johnson et al., 2015). Women consistently had a higher incidence of HZ than men (5.25 vs. 3.66 per 1,000 person-years), and the age- and sex-standardized incidence rate was 4.63 per 1,000 person-years (Johnson et al., 2015). Figure 2 shows age-specific incidence of HZ among immunocompetent individuals.

Recent population-based surveillance data further underscore the substantial burden of HZ and PHN among older adults. For example, a retrospective study reported that HZ and PHN were more common among adults aged ≥65 years, with PHN occurring in 26.4% of older patients compared with 15.3% of those aged < 65 years (Gudin et al., 2019). Marcum et al. (2024) reported that between 2019 and 2021, the standardized annual incidence of herpes zoster ranged from 542 to 685 cases per 100,000 person-years, while the incidence of post-herpetic neuralgia ranged from 35 to 38 cases per 100,000 person-years.

A cohort study reported that the incidence of HZ was 258.8 per 100,000 person-years among vaccinated individuals compared with 893.1 per 100,000 person-years among unvaccinated individuals (Sun et al., 2021). A Brazilian Delphi study reported that the proportion of HZ patients developing PHN increased with age, rising from 4% among adults aged 50–59 years to 14% among those aged ≥80 years (Bagattini et al., 2025). A regional workshop involving Indonesia, Malaysia, the Philippines, Thailand, and Vietnam identified substantial gaps in epidemiological data on HZ across Southeast Asia (Bibera et al., 2024). Available evidence indicated an increasing clinical and socioeconomic burden of HZ, particularly among older adults and individuals with immunocompromising conditions (Bibera et al., 2024).

A study in Japan reported standardized incidence rates of 9.58 and 1.00 per 1,000 person-years for HZ and PHN, respectively (Adachi-Katayama et al., 2026). Between 2014 and 2023, the incidence of both HZ and PHN increased significantly, with annual percent changes of 1.16% and 0.99%, respectively. The incidence of both conditions increased progressively with age, with the highest rates observed among adults aged ≥80 years (Adachi-Katayama et al., 2026).

In pregnancy, a study indicated that HZ occurred throughout pregnancy, with 26.4% of cases occurring in the first trimester, 36.1% in the second trimester, and 37.5% after 28 weeks of gestation (Xu et al., 2025). The thoracic dermatomes were most commonly affected (38.9%), and 98.6% of women experienced neuropathic pain (Xu et al., 2025).

#### 3.2.2 Clinical risk factors

In a population-based cohort study involving 119,413 patients with herpes zoster, 5.8% (6,956) developed PHN (Forbes et al., 2016a,b). The risk of PHN increased markedly with advancing age, particularly between 50 and 79 years, and was higher among women than men (Forbes et al., 2016a,b). Severe immunosuppressive conditions, including leukemia and lymphoma, autoimmune diseases such as rheumatoid arthritis, and comorbidities including asthma and diabetes mellitus were associated with an increased risk of PHN (Forbes et al., 2016a,b). Current and former smokers, as well as underweight and obese individuals, also had a higher risk of developing PHN. Antiviral therapy was not significantly associated with PHN incidence overall; however, it appeared to attenuate the increased risk among severely immunocompromised patients (Forbes et al., 2016a,b).

A Japanese study indicated that the risks of hospitalization and PHN following HZ were 4.8% and 10.4%, respectively, and were approximately 2–3 times higher among individuals aged ≥70 years than those aged 50–59 years (Adachi-Katayama et al., 2026).

Research indicates that individuals living with HIV infection, patients with hematological malignancies or solid organ cancers, recipients of organ or stem-cell transplantation, and those receiving corticosteroids, chemotherapy, biologic agents, or other immunosuppressive therapies have significantly higher incidence rates than the general population (Liang et al., 2025). Meta-analyses reported that chronic medical conditions including diabetes mellitus, chronic kidney disease, chronic obstructive pulmonary disease (COPD), rheumatoid arthritis, and systemic lupus erythematosus (SLE) have also been associated with increased susceptibility to herpes zoster (Liang et al., 2025; Wang et al., 2025).

### 3.3 Management of HZ and PHN

#### 3.3.1 Pharmacological management

Early antiviral therapy remains the cornerstone of acute HZ management. Current clinical guidelines recommend initiating antiviral treatment within 72 h of rash onset, although treatment may still be beneficial in patients presenting later if new vesicles continue to appear or if complications such as herpes zoster ophthalmicus or neurological involvement are present (Koshy et al., 2018). The primary antiviral agents include acyclovir, valacyclovir, and famciclovir, which inhibit viral DNA polymerase, thereby limiting viral replication, accelerating lesion healing, reducing the severity and duration of acute pain, and decreasing viral shedding (Paintsil and Cheng, 2019). Although antiviral therapy does not completely prevent PHN, early treatment reduces the severity of acute herpes zoster and may lower the risk of prolonged pain in selected patients (Chen et al., 2014).

A systematic review of six randomized controlled trials (1,211 participants) found that neither oral acyclovir nor famciclovir significantly reduced the incidence of PHN compared with placebo (Chen et al., 2014). Meta-analysis showed no significant reduction in PHN at 4 or 6 months following rash onset (Chen et al., 2014). Although acyclovir showed some evidence of reducing acute pain within the first 4 weeks, famciclovir did not significantly reduce the incidence of PHN. Both antiviral agents were generally well tolerated, with adverse event rates comparable to placebo (Chen et al., 2014).

A study on pregnant women with HZ reported that HZ was treated with oral acyclovir, topical penciclovir, calamine, or Radix isatidis. Severe cases with extensive lesions and intense pain may require antiviral therapy and appropriate analgesic management, with careful consideration of potential fetal risks and benefits (Xu et al., 2025).

Another study in Brazil reported that pain management among patients commonly included pregabalin, gabapentin, and codeine-based analgesics, reflecting current real-world treatment practices for HZ and PHN (Bagattini et al., 2025). Pain management during the acute phase should be individualized according to pain severity (Adriaansen et al., 2024). Mild pain may be adequately controlled with paracetamol or non-steroidal anti-inflammatory drugs (NSAIDs), whereas moderate-to-severe pain may require short-term opioid therapy under careful clinical supervision.

In a population-based study of 142,216 incident herpes zoster cases, 58.1% of patients received antiviral therapy, with aciclovir accounting for 69.0% of prescriptions (Forbes et al., 2012). Antiviral prescribing increased with age until 65 years but declined among patients aged ≥85 years (Forbes et al., 2012). Female sex, higher socioeconomic status, and immunosuppressive conditions were associated with a greater likelihood of receiving antiviral treatment, although antivirals were not prescribed routinely to all immunosuppressed patients (Forbes et al., 2012). A Brazilian study indicates that acyclovir and valaciclovir were the preferred antiviral agents in the public and private healthcare sectors, respectively (Bagattini et al., 2025).

Valacyclovir and famciclovir have demonstrated clinical efficacy comparable to acyclovir while offering simpler dosing schedules and improved oral bioavailability, which may enhance patient adherence (Bist et al., 2020). A Chinese study highlighted that these second-generation antiviral agents have increased steadily between 2010 and 2019 because of their favorable safety profile and effectiveness, particularly among women and adults aged ≥65 years (Yu et al., 2022). Similarly, findings emphasized that prompt antiviral therapy significantly improves acute clinical outcomes when initiated early in the course of disease (Sauerbrei, 2016).

Research indicates that corticosteroids may reduce acute pain 6 months after the onset of HZ and improve QoL in selected immunocompetent patients when used in combination with antiviral therapy; however, they should not be administered as monotherapy because they do not prevent PHN and may increase the risk of adverse effects in susceptible individuals (Jiang et al., 2023).

Management of PHN primarily targets neuropathic pain. Current evidence-based guidelines recommend gabapentin, pregabalin, tricyclic antidepressants (TCAs), and topical lidocaine as first-line therapeutic options (Sauerbrei, 2016; Attal et al., 2010). TCAs such as amitriptyline and nortriptyline inhibit the reuptake of serotonin and norepinephrine, thereby enhancing descending inhibitory pain pathways. Although these agents are effective, their anticholinergic adverse effects, including dry mouth, constipation, urinary retention, orthostatic hypotension, and sedation, may limit their use, particularly among older adults (Attal et al., 2010). Moore et al. reported that adverse events occurred more frequently among patients receiving amitriptyline than placebo, (Moore et al., 2015). while Derry et al. found limited evidence supporting the efficacy of nortriptyline and reported a higher incidence of treatment-related adverse effects (Derry et al., 2015). Consequently, treatment decisions should balance analgesic benefit against tolerability and patient comorbidities.

A study indicates that opioids were the most frequently prescribed initial treatment for PHN (21.6%), followed by gabapentin (15.1%), prescription NSAIDs (8.9%), and lidocaine patches (8.3%) (Gudin et al., 2019). However, only 24.6% of patients aged < 65 years and 38.5% of those aged ≥65 years received guideline-recommended first-line therapies (gabapentin, pregabalin, lidocaine patches, or tricyclic antidepressants) (Gudin et al., 2019). Patients initiated on opioid therapy also incurred the highest additional healthcare expenditures compared with HZ patients without PHN (Gudin et al., 2019).

Clinical studies have consistently demonstrated their effectiveness in reducing PHN-related pain and improving sleep quality and overall QoL (Tsubaki et al., 2024; Yu and Lu, 2025; Mehta et al., 2016). Dose adjustment is necessary in patients with renal impairment because both agents are primarily eliminated through the kidneys. For patients with persistent pain despite monotherapy, combination treatment may be considered. Gilron et al. (2009) demonstrated that combined gabapentin and nortriptyline therapy produced greater pain reduction than either agent alone.

Other opioid analgesics, including tramadol, morphine, and oxycodone, could also serve as second- or third-line options for patients with severe PHN who fail to respond adequately to first-line therapies or who have contraindications to other medications (McNicol et al., 2013). Topical therapies provide localized analgesia while minimizing systemic adverse effects and are particularly useful in older adults or patients with multiple comorbidities (Labianca et al., 2012; Leonardi et al., 2015; Noble et al., 2010). The 5% lidocaine patch is recommended as a first-line topical treatment because of its favorable safety profile, whereas the 8% capsaicin patch may be considered for patients with localized refractory pain following appropriate application under medical supervision (Derry et al., 2017; Tang and Zhao, 2026).

#### 3.3.2 Non-pharmacological management of post-herpetic neuralgia

Non-pharmacological interventions serve as valuable adjuncts to pharmacological therapy, particularly among patients with persistent pain, medication intolerance, or psychosocial impairment. Although the quality of evidence varies, these approaches may improve pain control, physical functioning, psychological wellbeing, and overall QoL.

Meta-analyses reveal that acupuncture has modest benefit in reducing neuropathic pain and improving functional outcomes (Wang et al., 2018; Zhou H. et al., 2021). Another study indicates that structured physical therapy and individualized exercise programmes may improve mobility, reduce physical disability, and enhance functional recovery in patients experiencing prolonged pain (Vasileiadis et al., 2022).

Again, psychological interventions, particularly cognitive behavioral therapy (CBT), relaxation, and sleep, help patients to cope with pain and mental health problems (Niemeyer et al., 2024). Research indicates additional adjunctive therapies include transcutaneous electrical nerve stimulation (TENS), peripheral nerve blocks, spinal cord stimulation, dorsal root ganglion stimulation, and other interventional pain procedures for carefully selected patients with refractory PHN (Wang et al., 2024; Isagulyan et al., 2023; Ing et al., 2015). Complementary therapies such as massage therapy, sound therapy, and selected herbal preparations have also been explored to treat PHN (Liang et al., 2017).

#### 3.3.3 Vaccination against herpes zoster

Vaccination represents the most effective strategy for preventing HZ and its complications, particularly PHN. The recombinant zoster vaccine (RZV; Shingrix^®^) is currently the preferred vaccine because of its high efficacy, durable immune protection, and favorable safety profile (Curran et al., 2019; Sun et al., 2021). A cohort study reported that Overall vaccine effectiveness was 85.5%, with effectiveness of 86.8% among adults aged 50–79 years and 80.3% among those aged ≥80 years with 2 doses of RZV (Shingrix^®^) (Sun et al., 2021). High vaccine effectiveness (84.8%) was also observed among individuals who had previously received the live zoster vaccine within the preceding 5 years (Sun et al., 2021).

In the ZOE-50 and pooled ZOE-70 clinical trials, the adjuvanted recombinant zoster vaccine (Shingrix^®^) demonstrated >90% efficacy in reducing the overall burden of HZ and its impact on daily activities (Curran et al., 2019). Among breakthrough HZ cases, vaccination significantly reduced pain severity, as measured by the Zoster Brief Pain Inventory (ZBPI), and showed a trend toward improved health-related QoL compared with placebo recipients (Curran et al., 2019).

A survey of 27 European Union countries found that 17 (63.0%) had national recommendations for HZ vaccination, although only 7 countries provided full public funding (Chiavarini et al., 2025). Vaccination was recommended for healthy adults aged ≥50 years in 23.5% of countries, ≥60 years in 29.4%, and ≥65 years in 41.2%. Nearly all countries (94.1%) also recommended vaccination for at-risk populations, most commonly from 18 years of age (Chiavarini et al., 2025). Another study also reported relatively low uptake of HZ vaccination. Between 1^st^ of January 2019 and 31^st^ of May 2022, among adults aged 50 years and older, 4.3% had received one dose of the recombinant zoster vaccine (Shingrix), 9.0% had completed the recommended two-dose series, whereas only 0.2% had received a single dose of the live attenuated zoster vaccine (Zostavax) (Marcum et al., 2024). In India, vaccination against HZ and PHN was noted as the most effective preventive strategy; however, noted that neither Zostavax^®^ nor Shingrix^®^ has been incorporated into India's national immunization programme (Varghese and Mishra, 2026).

Major barriers to vaccine uptake against HZ and PHN in LMICs included limited public awareness, high vaccine costs, and the absence of national surveillance data (Varghese and Mishra, 2026). A workshop in Asian communities also highlighted limited awareness among both healthcare professionals and the public, and inconsistencies in national vaccination recommendations (Bibera et al., 2024).

**Figure 3:**
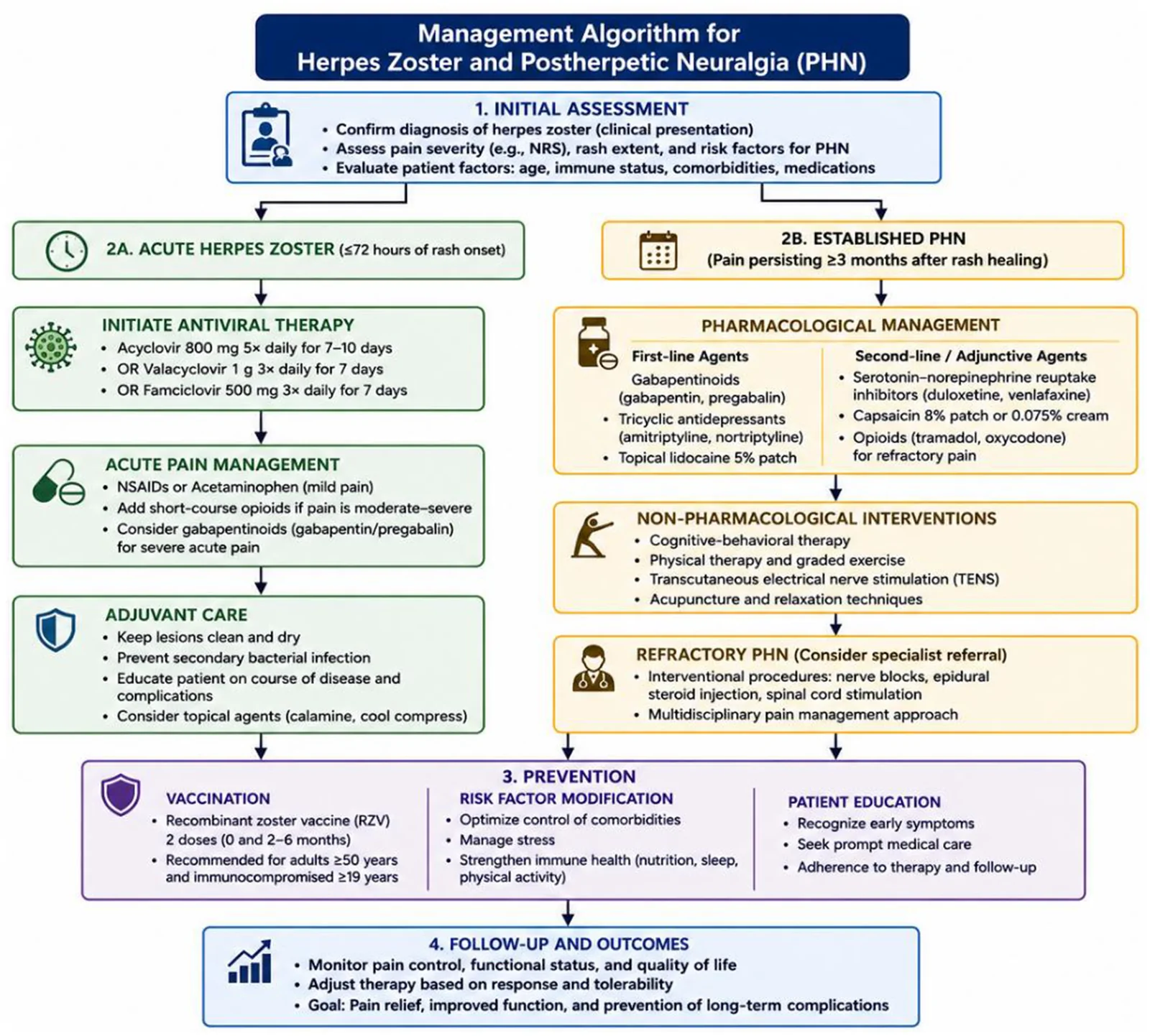
Evidence-based management algorithm for HZ and PHN.

Figure 3 shows an evidence-based management algorithm for HZ and PHN. The algorithm summarizes the clinical approach to assessment, early antiviral therapy, acute pain management, pharmacological and non-pharmacological treatment of PHN, preventive strategies including recombinant zoster vaccination, patient education, and follow-up to improve long-term outcomes.

### 3.4 Long-term outcomes of HZ and PHN

HZ and its most frequent complication, PHN, have been shown to impact QoL considerably and functional status (Liu Q. et al., 2024; Serpell et al., 2014; Drolet et al., 2010). A cohort study found that approximately 30% (189/633) of Chinese patients developed post-herpetic neuralgia (PHN) (Liu Q. et al., 2024). Among patients without PHN, pain severity measured using the Zoster Brief Pain Inventory (ZBPI) and QoL scores improved substantially, with near-complete resolution by 90 days after rash onset (Liu Q. et al., 2024). In contrast, patients who developed PHN showed persistent pain and sustained impairment in quality of life, with no significant improvement in ZBPI pain scores or quality-of-life measures by 180 days after rash onset (Liu Q. et al., 2024).

The UK-wide Zoster Quality of Life (ZQOL) study found that 59.9% of patients with PHN experienced moderate-to-severe pain (ZBPI worst pain ≥5) despite multiple consultations and treatment (Serpell et al., 2014). PHN was associated with significantly poorer health-related QoL compared with age-matched norms, with older patients experiencing greater pain severity and functional impairment, while many reported dissatisfaction with treatment effectiveness (Serpell et al., 2014).

Findings such as sleep, enjoyment of life, general activities, mood, normal work, and the QoL domains of pain or discomfort and usual activities were particularly affected across all age groups (Drolet et al., 2010). The most commonly mentioned issues among PHN individuals were anxiety or depression, pleasure in life, mood, and sleep (Drolet et al., 2010). Significant impairment in certain age groups suggested that younger patients had an equal risk of developing acute HZ. Still, PHN was more common in older individuals than in younger ones. As a result, older participants' QoL was more severely impaired of life, preventative actions might be especially helpful for certain age ranges (Drolet et al., 2010).

Previous studies assessed pain and discomfort caused by HZ using the Zoster Brief Pain Inventory (ZBPI) (Matthews et al., 2023; Curran et al., 2018; Sato et al., 2017). This tool measures pain, different types of discomfort (such as itching) at the rash site, and how HZ affects seven daily activities: work, sleep, walking, enjoyment of life, mood, relationships with others, and general activities (Drolet et al., 2010).

Another tool, the Zoster Impact Questionnaire (ZIQ), assesses how HZ pain and PHN affect the functional aspects of life (Johnson et al., 2010). As HZ and PHN have an impact on our daily activities, both of these tools are considered sensitive and accurate (Johnson et al., 2010; Matthews et al., 2023; Curran et al., 2018; Sato et al., 2017). The majority of individuals suffering from acute HZ injury report moderate to severe dermatomal pain in the skin.

The cause of acute-phase pain is the damage brought by viral replication to neural tissues and defense mechanisms (Forstenpointner et al., 2018). Patients rate their level of pain during the acute stage as being more severe than recovery or labor pain (Xu et al., 2025). PHN is characterized by constant and/or intermittent paroxysmal pain persisting for ≥90 days after the onset of HZ rash (Ngo et al., 2020). Approximately 70% of patients have allodynia, which is widely regarded as the most painful and incapacitating aspect of PHN (Xu et al., 2023). An additional factor in the burden of PHN is numbness, tingling, and “Neuropathic Pain,” which may sustain injuries if patients scrape their skin until the protective feeling is lost (Dai et al., 2026). PHN pain induces a negative impact on the lives of patients and causes a decline in general health, a loss of physical function, including weariness, anorexia, weight loss, inactivity, insomnia, loss of social contact, withdrawal, and isolation because patients experience reduced independence, withdraw from social activities, and participate less in social gatherings (Johnson et al., 2010).

Patients also develop functional limitations that can restrict mobility and participation in regular activities, leading to physical decline and increased risk of related health issues. One study found that, compared with patients with HZ, those with PHN experienced significantly greater discomfort that interfered with their ability to walk (P < 0.05) (Weinke et al., 2010). However, multiple regression analysis showed no significant association between walking ability and QoL scores.

Mobility issues were reported by a large percentage of patients (63%), especially those with PHN (Weinke et al., 2010). Mobility was severely limited in 19% of PHN patients, who were confined to bed (Weinke et al., 2010).

Significant differences were observed across all temperament domains, with HZ patients predominantly exhibiting a hyperthymic temperament and PHN patients primarily exhibiting an anxious temperament (Akkemik and Yigit Tekkanat, 2026). PHN patients also had significantly higher depressive, cyclothymic, irritable, and anxious temperament scores, with large effect sizes. Furthermore, depression and anxiety were more severe and quality of life was poorer among PHN patients. Strong correlations were reported between temperament dimensions and pain intensity, particularly among PHN patients, as well as between temperament dimensions and psychological measures (Akkemik and Yigit Tekkanat, 2026). Complications can be ophthalmic (for example, herpes zoster ophthalmicus), neurological (for example, cranial and peripheral nerve palsies), visceral (for example, pneumonia), dermatological (for example, bacterial superinfection), and cervical and lumbosacral injuries (Yawn et al., 2013).

A Brazilian study reveals that ophthalmic and neurological complications occurred in 5–13% of patients, while hospitalization rates ranged from 1%−8% (Bagattini et al., 2025). Available evidence also demonstrated substantial healthcare resource utilization, with most patients requiring 2–3 medical consultations, frequent specialist referrals, and considerable work absenteeism, highlighting the increasing clinical and economic burden of HZ and PHN among older adults (Bagattini et al., 2025).

There is also an increased risk of paresis and stroke (Haanpää, 2017). Patients with acute HZ are also seen with severe, common, debilitating chronic conditions such as congestive heart failure (CHF), diabetes mellitus (DM), myocardial infarction (MI), and clinical depression. As patients age, the frequency of complications increases: those over 65 have four times the number of complications compared to people under the age of 35 years (Johnson et al., 2010). Most pregnancies affected by HZ resulted in favorable outcomes, with the majority of infants delivered at term and having normal birth weights and excellent Apgar scores (Xu et al., 2025). However, rare adverse outcomes, including miscarriage, stillbirth, preterm birth, and neonatal anomalies, were reported. Severe maternal disease may also have persistent consequences, as illustrated by a case of extensive HZ complicated by severe keloid formation and post-herpetic neuralgia requiring long-term pregabalin therapy (Xu et al., 2025).

The long-term outcomes of HZ and PHN can profoundly affect both physical health and emotional wellbeing. While some individuals recover without lasting issues, others face significant challenges that require ongoing support and management. Vaccination can help prevent the onset and severity of these conditions, particularly in older adults.

## 4 Discussion

### 4.1 Major insights on epidemiology and management strategies

The reviewed literature indicates that the incidence of PHN increases markedly with advancing age, affecting up to one-third of patients aged 80 years and older. The incidence of HZ and PHN found in this study was consistently higher among women, older adults, and immunocompromised individuals, reflecting the influence of age-related decline in cell-mediated immunity and underlying immune dysfunction (Li et al., 2026). The reviewed literature indicates the persistent burden of HZ and PHN despite the availability of highly effective vaccines and emphasizes the need to improve adult vaccination coverage, particularly among populations at highest risk of disease and its complications (Marcum et al., 2024). This review also identified several notable risk factors for the development of PHN. Advanced age, particularly individuals over the age of 50, is at significantly higher risk compared to younger patients (Ogunyemi et al., 2025; Borkar et al., 2013; Forbes et al., 2016a). This demographic vulnerability highlights the need for targeted public health initiatives for older adults, including education on shingles prevention and the importance of vaccination. Additionally, the severity of the initial HZ infection plays a crucial role, with those experiencing more intense acute pain and a more severe rash outbreak more likely to progress to chronic neuropathic pain (Adriaansen et al., 2024; Forbes et al., 2016a; Zhou Q. et al., 2021). Recognizing these factors can lead to improved screening and early intervention strategies, ultimately reducing the burden of chronic pain in the aging population. The presence of prodromal pain before the onset of the rash has also been associated with an increased risk of PHN (Forbes et al., 2016a).

Although HZ occurs in all geographical regions, epidemiological patterns vary according to population age structure, healthcare access, surveillance systems, and vaccination policies. Most incidence data originate from North America, Europe, Japan, South Korea, and Australia, where aging populations have contributed to increasing disease burden over recent decades (Chen et al., 2025). In contrast, epidemiological data from many LMICs remain limited under-reporting, inconsistent surveillance systems, and limited access to laboratory confirmation (Wu et al., 2025).

The influence of universal childhood varicella vaccination on HZ epidemiology was extensively investigated in the present study. Early mathematical models suggested that reduced circulation of wild-type VZV might temporarily decrease opportunities for exogenous immune boosting among adults, potentially increasing HZ incidence (Park and Byeon, 2026). However, subsequent population-based studies have produced inconsistent findings. The reviewed literature indicates that demographic aging, improved case recognition, greater healthcare utilization, and increasing numbers of immunocompromised individuals are likely to be the principal contributors to observed increases in HZ incidence in many settings rather than childhood varicella vaccination alone (Marijam et al., 2024; Cho et al., 2026).

One of the most clinically important consequences of HZ is PHN, the most frequent chronic complication of the disease. Approximately 10%−20% of patients with HZ develop PHN, although reported estimates vary depending on the definition used, study population, and duration of follow-up (Johnson et al., 2010). PHN is associated with persistent neuropathic pain, sleep disturbance, depression, reduced physical functioning, impaired QoL, and increased healthcare utilization, making it one of the major contributors to the overall burden of HZ (Curran et al., 2023).

Hospitalization and mortality attributable to HZ found in this review are relatively uncommon among immunocompetent younger adults but increase substantially among older adults and immunocompromised patients (Li et al., 2026). Severe complications such as herpes zoster ophthalmicus, disseminated herpes zoster, bacterial superinfection, meningitis, encephalitis, stroke, and cranial nerve palsies contribute to increased morbidity and healthcare costs (Akkemik and Yigit Tekkanat, 2026; Haanpää, 2017; Borkar et al., 2013). The economic burden includes direct medical costs associated with outpatient visits, hospitalization, antiviral treatment, pain management, and long-term care, as well as indirect costs resulting from work absenteeism and reduced productivity (Chaiyakunapruk et al., 2024). The available evidence suggests that previously available **Zostavax**^®^ demonstrated moderate effectiveness but significantly lower efficacy among older adults, with protection declining over time. For this reason, recombinant zoster vaccine has largely replaced Zostavax in countries where it is available (Oleszko et al., 2025). Clinical trials and real-world effectiveness studies have consistently demonstrated vaccine efficacy exceeding 90% against HZ and PHN among immunocompetent older adults, with substantial protection maintained over several years (Oleszko et al., 2025; Mbinta et al., 2026).

In terms of management strategies, early intervention has emerged as a crucial component in mitigating the risk of PHN. Prompt initiation of antiviral medication, such as acyclovir, valacyclovir, or famciclovir, within 72 h of the HZ rash can help reduce the likelihood of transitioning to chronic pain (Kim et al., 2021; Schutzer-Weissmann and Farquhar-Smith, 2017). This emphasizes the necessity for public health initiatives that ensure accessibility to antiviral treatments and educate patients on the importance of early treatment. Similarly, aggressive pain control during the acute phase, utilizing a combination of oral analgesics, topical treatments, and adjuvant therapies, has been linked to a lower incidence of PHN development (Kim et al., 2021). This not only improves QoL but also reduces the burden on healthcare systems by minimizing long-term complications associated with untreated HZ.

The reviewed literature indicates the effectiveness of Shingrix in reducing HZ incidence among older adults, including those with chronic medical conditions and selected immunocompromised populations, especially in European countries (Chiavarini et al., 2025). Consequently, many national and international immunization guidelines recommend recombinant zoster vaccination for adults aged ≥50 years and for younger individuals at increased risk because of immunosuppression (Curran et al., 2019).

The reviewed literature indicates that two doses of Shingrix have demonstrated vaccine efficacy exceeding 90% against both HZ and PHN in immunocompetent adults aged 50 years and older, with sustained protection for several years following vaccination (Oleszko et al., 2025).

This review also indicates the importance of a multidisciplinary approach to managing PHN. The use of gabapentinoids, tricyclic antidepressants, and topical lidocaine has all demonstrated efficacy in alleviating the neuropathic pain associated with this condition. This study suggests that carefully selected combination regimens may benefit patients with refractory PHN. Additionally, non-pharmacological interventions, such as physical therapy, CBT, and interventional pain management techniques, have shown promise in improving patient outcomes by reducing pain catastrophizing, improving emotional wellbeing, and addressing anxiety or depression associated with chronic herpetic pain (Forstenpointner et al., 2018; Pei et al., 2019). In this review, acupuncture was found to be beneficial in the treatment of PHN; however, larger, high-quality randomized controlled trials are required to confirm its long-term effectiveness (Wang et al., 2018; Zhou H. et al., 2021). Herbal supplements were found to be effective for hepatic pain; however, current evidence remains limited, and further well-designed clinical trials are required before these interventions can be routinely recommended (Jeon et al., 2022).

### 4.2 Best practices for management and prevention

#### 4.2.1 Evidence-based recommendations and guidelines for patient care for clinicians

Several professional organizations have developed evidence-based clinical practice guidelines for the management of PHN (Henze et al., 2022). The most recent guidelines from the Neuropathic Pain Special Interest Group of the International Association for the Study of Pain (NeuPSIG) (Truini et al., 2023), the UK National Institute for Health and Care Excellence (NICE) (NICE, 2020), the European Federation of Neurological Societies (EFNS) (Attal et al., 2010), the Canadian Pain Society (CPS) (Moulin et al., 2014) and expert panels from South Africa (Chetty et al., 2013). and the Middle East (Bohlega et al., 2010)., recommend a patient-centered, stepwise approach to neuropathic pain management, emphasizing individualized treatment, regular monitoring, and specialist referral for severe or refractory cases.

First-line therapies include amitriptyline, duloxetine, gabapentin, and pregabalin, with treatment modification based on efficacy and tolerability. Short-term tramadol or topical capsaicin may be used in selected patients, while carbamazepine remains the preferred first-line treatment for trigeminal neuralgia. Long-term opioid use and several other agents are discouraged unless recommended by a specialist, and careful consideration of drug safety, including pregnancy risks and misuse potential, is advised (Truini et al., 2023; NICE, 2020; Moulin et al., 2014; Chetty et al., 2013; Bohlega et al., 2010).

In the revised guidelines of the Neuropathic Pain Special Interest Group of the International Association for the Study of Pain (2015) (Truini et al., 2023)., the first-line treatments recommended include gabapentin, pregabalin, serotonin-norepinephrine reuptake inhibitors (SNRIs) such as duloxetine and venlafaxine, and tricyclic antidepressants (TCAs). Gabapentin and pregabalin are widely recommended because they reduce neuronal hyperexcitability through binding to the α2δ subunit of voltage-gated calcium channels, thereby decreasing the release of excitatory neurotransmitters involved in neuropathic pain (NICE, 2020).

The NICE guideline recommends a patient-centered approach to managing neuropathic pain in non-specialist settings, emphasizing regular assessment, individualized treatment, and ongoing monitoring (NICE, 2020). First-line pharmacological options include amitriptyline, duloxetine, gabapentin, and pregabalin, with treatment adjusted according to efficacy and tolerability. Short-term tramadol and topical capsaicin cream may be considered in selected patients, while carbamazepine remains the first-line treatment for trigeminal neuralgia (NICE, 2020). The guideline also highlights the importance of specialist referral for severe or refractory pain and cautions against initiating several medications, including long-term opioids, without specialist advice due to limited benefit or safety concerns (NICE, 2020).

European Federation of Neurological Societies revealed that gabapentin, pregabalin, or a TCA are recommended as first-line therapies. For elderly patients, where systemic side effects of oral medications are a concern, topical lidocaine is permitted as a first-line option. Strong opioids and topical capsaicin (0.075%) are recommended as second-line treatments (Attal et al., 2010).

High-concentration capsaicin patches have demonstrated moderate efficacy in reducing PHN pain but are commonly associated with transient local burning and erythema. Lidocaine patches have also shown significant benefit with minimal systemic toxicity, making them especially valuable for frail or elderly patients (Santos et al., 2024).

The updated guidelines of the Canadian Pain Society (2014) suggest gabapentin, pregabalin, duloxetine, venlafaxine, and TCAs as first-line therapies. Second-line options include tramadol, strong opioids, and lidocaine cream or patches, while cannabinoids may be considered as a third-line alternative (Moulin et al., 2014).

South African Expert Panel (Chetty et al., 2013) identifies pregabalin, gabapentin, or amitriptyline as first-line therapies. For second-line treatment, a combination of first-line agents from different classes is recommended. Opioids, beginning with tramadol, and then stronger opioids, are reserved for third-line therapy, while the Middle Eastern Expert Panel (Bohlega et al., 2010) guideline recommends pregabalin, gabapentin, and topical lidocaine (specifically for focal PHN with allodynia) as first-line treatment options. SNRIs and opioids, including tramadol, are classified as second-line options according to the guideline. These guidelines are summarized in Table 1.

**Table 1:** Comparison of international and regional guideline recommendations for the pharmacological management of herpes zoster–associated neuropathic pain and post-herpetic neuralgia.

Guideline/ association	First-line therapies	Second-line therapies	Third-line/alternative therapies
NICE (2020)	Amitriptyline, duloxetine, gabapentin, pregabalin; carbamazepine for trigeminal neuralgia	Switch to another first-line agent; topical capsaicin cream for localized pain	Short-term tramadol (rescue therapy); specialist referral for refractory pain; avoid routine long-term opioids and other non-recommended agents
NeuPSIG (IASP, 2015)	TCAs, SNRIs (duloxetine, venlafaxine), gabapentin, pregabalin	Lidocaine 5% patch, capsaicin 8% patch, tramadol	Strong opioids (morphine, oxycodone), botulinum toxin A
American academy of neurology (AAN)	Pregabalin, gabapentin	Amitriptyline, topical lidocaine patch	Capsaicin and opioids for selected refractory patients
European academy of neurology (EAN/EFNS)	TCAs, pregabalin, gabapentin, duloxetine	Lidocaine 5% patch, capsaicin 8% patch	Strong opioids under specialist supervision
Canadian pain society (2014)	TCAs, gabapentin, pregabalin, duloxetine	Tramadol, topical lidocaine	Strong opioids, cannabinoids, methadone in selected patients
South African expert consensus (2010)	Early antiviral therapy (acyclovir, valacyclovir, famciclovir) for acute HZ; gabapentin, pregabalin, TCAs for PHN	Topical lidocaine or capsaicin; tramadol for inadequate pain control	Strong opioids, interventional pain procedures, and specialist referral for refractory PHN
Middle East expert consensus (2010)	Prompt antiviral therapy within 72 h of rash onset; gabapentin, pregabalin, TCAs, duloxetine for PHN	Topical lidocaine or capsaicin; tramadol as add-on therapy	Strong opioids, combination therapy, nerve blocks, and referral to pain specialists for persistent or severe PHN

AAN, American academy of neurology; EAN, European academy of neurology; EFNS, European federation of neurological societies; HZ, herpes zoster; IASP, international association for the study of pain; NICE, national institute for health and care excellence; NeuPSIG, neuropathic pain special interest group; PHN, post-herpetic neuralgia; SNRI, serotonin–norepinephrine reuptake inhibitor; TCA, tricyclic antidepressant.

Overall, pharmacological management should be individualized based on patient age, pain severity, comorbidities, medication tolerability, and treatment response. A multimodal approach combining pharmacological and non-pharmacological interventions often provides the greatest improvement in pain control, functional status, and health-related QoL.

## 5 Implications of clinical practice

The findings from the study on PHN underscore the importance of early recognition, prompt treatment, and a comprehensive management approach. By integrating these insights into patient care strategies, clinicians can work to mitigate the risk of PHN development, improve symptom control, and enhance the overall quality of life for patients affected by this challenging condition. Furthermore, considering personalized treatment plans, individual risk factors, and patient preferences may help optimize outcomes and facilitate a more tailored approach to managing PHN. Vaccination barriers underscore the need for community-based epidemiological studies and strengthened public health initiatives to improve vaccination coverage. Ongoing research and implementing best practices can contribute to the continued improvement of care for individuals suffering from PHN.

## 6 Strengths and limitations

### 6.1 Strengths of the review

This review has several notable strengths. First, it provides a comprehensive synthesis of recent evidence on the epidemiology, risk factors, pathogenesis, clinical manifestations, management, prevention, and long-term outcomes of HZ and PHN. Second, it integrates findings from diverse populations and clinical settings, offering a broad perspective on the current global burden and evolving management strategies. Third, the review critically compares international guideline recommendations and summarizes evidence supporting pharmacological and non-pharmacological interventions, facilitating evidence-based clinical decision-making. Fourth, the inclusion of illustrative figures and summary tables enhances the presentation of complex concepts, making the review more accessible to clinicians, researchers, and healthcare trainees. Finally, by identifying persistent knowledge gaps, including vaccine uptake, management challenges in resource-limited settings, and the need for high-quality comparative studies, this review provides direction for future research and policy development.

### 6.2 Limitations

This review has several limitations. First, the literature search was restricted to studies published between January 2010 and June 2026; therefore, newer evidence may modify the current conclusions. Second, only English-language, published studies were included, introducing potential language and publication bias through the exclusion of non-English and gray literature. Third, although the review comprehensively examined clinical, demographic, and environmental risk factors, it did not extensively evaluate genetic factors, biomarkers, or the influence of comorbidities on HZ and PHN outcomes. In addition, the exclusion of pediatric populations limits the generalizability of the findings to children and adolescents. Future research should incorporate updated evidence, include non-English and gray literature, investigate genetic and biomarker-based predictors, evaluate the role of comorbidities, and expand the evidence base to include pediatric populations

## 7 Conclusion

This review underscores the complexity of HZ and PHN etiopathogenesis and the significant burden they impose, particularly on older adults. Key findings highlight that risk factors such as advanced age, severity of the initial HZ rash, and immunocompromised status markedly increase the likelihood of developing PHN. Early intervention with antiviral treatments within 72 h of rash onset is crucial in reducing the risk of chronic pain. By addressing these areas, future research can significantly advance the understanding and treatment of HZ and PHN, ultimately leading to improved patient care and quality of life.

## Data Availability

The original contributions presented in the study are included in the article/supplementary material, further inquiries can be directed to the corresponding author.
